# Noise-Induced Changes of the Auditory Brainstem Response to Speech—a Measure of Neural Desynchronisation?

**DOI:** 10.1007/s10162-020-00750-7

**Published:** 2020-04-13

**Authors:** Jessica de Boer, Helen E. Nuttall, Katrin Krumbholz

**Affiliations:** 1 Hearing Sciences, Division of Clinical Neuroscience, School of Medicine, University of Nottingham, Science Road, University Park, Nottingham, NG7 2RD UK; 2 Department of Psychology, Lancaster University, Lancaster, UK

**Keywords:** auditory temporal processing, cochlear dispersion, subcortical auditory-evoked potentials, speech-in-noise difficulty, complex-ABR (cABR)

## Abstract

It is commonly assumed that difficulty in listening to speech in noise is at least partly due to deficits in neural temporal processing. Given that noise reduces the temporal fidelity of the auditory brainstem response (ABR) to speech, it has been suggested that the speech ABR may serve as an index of such neural deficits. However, the temporal fidelity of ABRs, to both speech and non-speech sounds, is also known to be influenced by the cochlear origin of the response, as responses from higher-frequency cochlear regions are faster and more synchronous than responses from lower-frequency regions. Thus, if noise caused a reweighting of response contributions from higher- to lower-frequency cochlear regions, the temporal fidelity of the aggregate response should be reduced even in the absence of any changes in neural processing. This ‘place mechanism’ has been demonstrated for non-speech ABRs. The aim of this study was to test whether it also applies to speech ABRs. We used the so-called ‘derived-band’ method to isolate response contributions from frequency-limited cochlear regions. Broadband and derived-band speech ABRs were measured both in quiet and in noise. Whilst the noise caused significant changes to the temporal properties of the broadband response, its effects on the derived-band responses were mostly restricted to the response amplitudes. Importantly, the amplitudes of the higher-frequency derived-band responses were much more strongly affected than those of the lower-frequency responses, suggesting that the noise indeed caused a reweighting effect. Our results indicate that, as for non-speech ABRs, the cochlear place mechanism can represent a potentially substantial confound to speech-ABR-in-noise measurements.

## Introduction

The ability to understand speech in noise (SiN) is often degraded as a result of hearing loss (Festen and Plomp 1990; Smoorenburg 1992) or in consequence of ageing (Dubno et al. 1984; Helfer and Wilber 1990), but can also be impaired in younger people and when hearing is clinically normal (Guest et al. 2018; Hind et al. 2011; Hope et al. 2013; Pienkowski 2017). The mechanisms underlying SiN deficits, particularly in the absence of hearing loss, are still poorly understood, but there is accumulating evidence that they can manifest in the auditory brainstem response (ABR) elicited by simple speech sounds (referred to as ‘speech ABR’). The speech ABR is typically evoked by a synthetic consonant-vowel syllable, /da/ (Anderson and Kraus 2010; Johnson et al. 2005), and consists of a series of peaks, which broadly follow the stimulus waveform and are thought to reflect synchronised neural responses from the rostral brainstem. When the syllable is presented in noise, the response peaks tend to be reduced in amplitude, increased in latency and broadened in shape. As a result, the stimulus-to-response correlation, which measures the degree of time locking between the response and stimulus waveform, is reduced, and the composition of the response frequency spectrum, which relates to the response shape, is altered. Some of these measures were sometimes found to be associated with SiN performance. In particular, several studies have reported an association between SiN performance and the latencies of some of the speech ABR peaks, with poorer SiN performance generally associated with longer peak latencies (Cunningham et al. 2001; Parbery-Clark et al. 2009; Anderson et al. 2010; Hornickel et al. 2011; Parbery-Clark et al. 2011; see, however, de Boer et al. 2012). Similarly, some studies have reported an association of SiN performance with the stimulus-to-response correlation of the speech ABR (Cunningham et al. 2001; Parbery-Clark et al. 2009, 2011, 2012), and some studies have found an association with the fundamental frequency and/or higher harmonics of the response frequency spectrum (Cunningham et al. 2001; Anderson et al. 2011; Song et al. 2011; Strait et al. 2012).

All studies have interpreted the observed noise effects on the speech ABR in terms of changes in neural temporal processing, assuming that noise degrades the synchrony of the neural responses to the speech stimulus and that this leads to the observed slowing and blurring of the resulting ABR. This interpretation is supported by findings that some speech ABR properties can be enhanced through auditory training (Anderson et al. 2013; Russo et al. 2005; Song et al. 2012; Parbery-Clark et al. 2013).

However, previous research on ABRs elicited by non-speech sounds, such as clicks and tone pips, has shown that noise effects on ABRs can also be influenced by a cochlear—and thus pre-neural—mechanism, referred to as the ‘place mechanism’ (Burkard and Hecox 1983b). A place effect arises when the noise causes a reweighting of response contributions from higher- to lower-frequency cochlear regions. Due to the cochlear travelling-wave delay (Elberling et al. 2007; Robles and Ruggero 2001), response contributions from lower-frequency regions are slower and less synchronised than higher-frequency responses (Don and Eggermont 1978; see Nuttall et al. 2015, for an equivalent finding on the speech ABR), and so, reweighting will cause ABR peaks to increase in latency, decrease in amplitude and broaden in shape. This place effect was substantial for stimuli with predominantly low-frequency content (1000-Hz tone pips; Burkard and Hecox 1983b), suggesting that it might also affect the response to the /da/ syllable, which too consists predominantly of low frequencies.

If this is the case, then previous interpretations of noise-induced changes in speech ABR properties purely in terms of neural mechanisms may be confounded. The current study aimed to test this. Using the so-called ‘derived-band’ method to isolate response contributions from limited cochlear regions (Teas et al. 1962), we measured both broadband and band-limited ABRs to a synthetic /da/ syllable similar to that used in previous studies. The syllable was presented both in quiet and in two levels of ‘uniformly exciting’ noise, that is noise designed to cause equally strong activation across all cochlear frequency regions (Glasberg and Moore 2000). Our results indicate that, as for non-speech ABRs, the place mechanism can substantially contribute to noise effects on the speech ABR, highlighting the need to control for this potential confound.

## Materials and Methods

### Participants

Twelve native English speakers (age range 18–39 years; mean age 21.2 years; 4 males) participated in this study after having given written informed consent. All had pure-tone hearing thresholds at or below 20 dB HL at octave frequencies between 250 and 8000 Hz bilaterally and reported no history of audiological or neurological disease. Participants were seated on a comfortable chair inside an electrically shielded, sound-attenuating booth (Industrial Acoustics Company, Winchester, UK). The experimental procedures complied with the Declaration of Helsinki guidelines (Version 6, 2008), but were not formally pre-registered online as set out in the Declaration’s 2014 amendment. They were approved by the Ethics Committee of the University of Nottingham Medical School. A subset of the current data, comprising all conditions measured in quiet, were included in a previous publication (Nuttall et al. 2015).

### Experimental Protocol

**Fig. 1:**
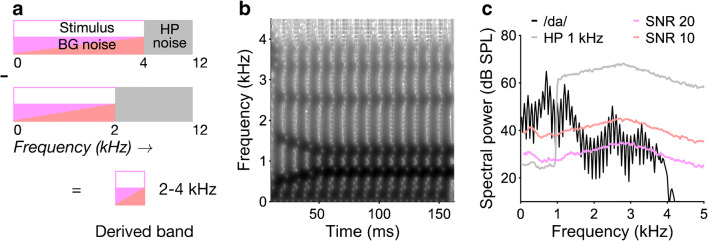
Experimental stimuli and derived-band procedure. **a** Schematic representation of the derived-band recording procedure, which involves subtracting high-passmasked responses for successive high-pass (HP) cutoff frequencies (2 and 4 kHz in this example). The HP noise is shown by the grey highlight, and the background (BG) noise is shown by the pink and red highlights (representing the two noise levels used). **b** Spectrogram of the /da/ syllable. The abscissa shows time, the ordinate shows frequency, and spectral energy is shown by the grey scale (with darker greys indicating increased energy). **c** Overall spectra of experimental stimuli as delivered through the ER-1 insert earphones used. The black line shows the /da/ syllable, the grey line shows an example high-pass noise with a 1-kHz cutoff frequency, and the pink and red lines show the full-spectrum background noise at the two signal-to-noise ratios (SNRs) used (20 and 10 dB, respectively). When presented in conjunction with a high-pass noise, the background noise would be low-pass-filtered at the HP cutoff frequency

Speech ABRs were elicited by a 170-ms synthetic consonant-vowel (/da/) syllable, which was presented either on its own to yield a ‘broadband’ speech ABR similar to the speech ABRs measured in previous studies or paired with a high-pass noise masker designed to eliminate response contributions above the high-pass cutoff frequency. Five different cutoff frequencies were used, spaced at octave intervals (0.5, 1, 2, 4 and 8 kHz). By subtracting the responses for successive cutoff frequencies, it is possible to isolate response contributions from the intervening octave frequency bands (i.e. 0.5–1, 1–2, 2–4 and 4–8 kHz; see Fig. 1a for a schematic example). These are the so-called ‘derived-band’ responses (see also Teas et al. 1962; Don and Eggermont 1978). In the current data, the 0.5–1- and 4–8-kHz responses were too noisy to analyse, and so, we instead used the 1-kHz high-pass-masked response, reflecting response contributions from all frequencies below 1 kHz (< 1-kHz band), and the difference between the broadband and 4-kHz high-pass masked responses, reflecting response contributions from all frequencies above 4 kHz (> 4-kHz band).

The broadband and high-pass masked responses were recorded both in quiet and in two levels of uniformly exciting noise (Glasberg and Moore 2000), yielding a total of 18 experimental conditions. The background noise levels corresponded to signal-to-noise ratios (SNRs) of 20 and 10 dB, respectively, which pilot tests had shown to be effective in causing notable changes in the broadband response properties, whilst at the same time not completely obliterating the response.

For each condition, we recorded two ‘replicate’ responses consisting of 2000 trials each. The recordings were distributed over two sessions lasting ~ 2 h each, conducted on different days spaced by no more than a week. For a given high-pass masking condition (including the broadband condition), all three background noise conditions were recorded on the same day. The order of conditions was counter-balanced across participants.

### Stimuli

The synthetic /da/ syllable was provided by Prof. Nina Kraus’s group at the Northwestern University, Chicago, USA, using a KLATT synthesiser. After an initial 10-ms stop burst, it was voiced throughout the rest of its 170-ms duration, with a constant 100-Hz fundamental frequency and five formant resonances. The stop burst was followed by a 50-ms formant transition and a 110-ms steady-state vowel. During the formant transition, the first formant rose from 400 to 720 Hz, and the second and third formants fell from 1700 to 1240 and 2580 to 2500 Hz, respectively, all with linear trajectories in linear frequency units. The fourth and fifth formants remained constant at 3300 and 3900 Hz, respectively (see Fig. 1b for the stimulus spectrogram).

Both the high-pass and background noises were derived from uniformly exciting noise, that is noise filtered to contain equal energy within all auditory filter bandwidths (defined in units of normal equivalent rectangular bandwidth, or ERB_N_; Glasberg and Moore 1990). To generate the high-pass noises, uniformly exciting noise was high-pass-filtered at the relevant cutoff frequency (0.5, 1, 2, 4 or 8 kHz), and low-pass-filtered at 12.2 kHz. For the corresponding background noises, uniformly exciting noise was low-pass-filtered at the relevant high-pass cutoff frequency (0.5, 1, 2, 4 or 8 kHz), or at 12.2 kHz when no high-pass noise was presented (i.e. in the broadband condition). All filtering was performed in the frequency domain. High-pass and low-pass filters were implemented as box-car filters (with infinitely steep slopes).

All stimuli were generated digitally at a 24.4-kHz sampling rate using MATLAB R2018a (The MathWorks, Natick, MA, USA), digital-to-analogue-converted with a 24-bit amplitude resolution using a TDT System 3 (Tucker Davis Technologies, Alachua, FL, USA) consisting of a real-time DSP processor (RP2.1) and a headphone amplifier (HB7) and presented monaurally to the left ear via ER-1 insert earphones (Etymotic Research Inc., Elk Grove Village, IL, USA). The speech syllable was presented at an overall sound pressure level (SPL) of 70 dB. The high-pass and background noises were presented at a fixed level per ERB_N_ within their passbands. For the high-pass noises, the level per ERB_N_ was set to 10 dB above the syllable level (70 + 10 = 80 dB SPL), and for the background noise, it was set to either 20 or 10 dB below the syllable level (70–20/10 = 50/60 dB SPL, respectively). The sound system was calibrated using an artificial ear (type 4153, Bruel & Kjaer UK Ltd., Royston, UK) connected to a DB2012 earphone adaptor (External Ear Simulator) and Sinus Apollo Light interface, operated with the Samurai noise and vibration measurement software (SINUS Messtechnik GmbH, Leipzig, Germany). Figure 1c shows the measured spectra of the syllable, the background noise at 20- and 10-dB SNR and an example high-pass noise with a 1-kHz cutoff frequency.

Both the high-pass and background noises were presented continuously throughout a given block of trials. The speech syllable was presented with alternating polarity to minimise stimulus artefacts. The syllable repetition rate was 3.3/s.

### ABR Recordings

ABRs were recorded with the clinical Smart EP system by Intelligent Hearing Systems (Miami, FL, USA) using the ‘electric-ABR’ mode, which allows the use of external stimuli and triggers. Neuroelectric signals were differentially measured between Ag-AgCl scalp electrodes placed at the vertex (Cz; positive) and the contralateral (right) earlobe (negative). An electrode placed on the mid-forehead (Fpz) served as common ground. Throughout the experiment, electrode impedances were maintained below 5 kΩ. The raw electrode signals were amplified by a factor of 10^5^ and bandpass-filtered online between 30 and 3000 Hz. A trigger at the beginning of each stimulus presentation initiated a 200-ms acquisition epoch. Epochs that exceeded ± 35 μV were rejected as artefactual. Non-artefactual epochs were averaged online (the Smart EP system does not allow saving individual epochs) until the average comprised 2000 epochs (1000 for each stimulus polarity), and the resulting average responses were analogue-to-digital-converted at a 20-kHz rate. Two such averages, *y*_1_ and *y*_2_, referred to as ‘replicates’, were recorded for each condition. The average of the two replicates, *y* = (*y*_1_ + *y*_2_)/2, was taken as a measure of the overall response, and the plus-minus reference, *e* = (*y*_1_ − *y*_2_)/2, was taken as a measure of the associated noise floor (Schimmel 1967).

### Data Analysis

**Fig. 2:**
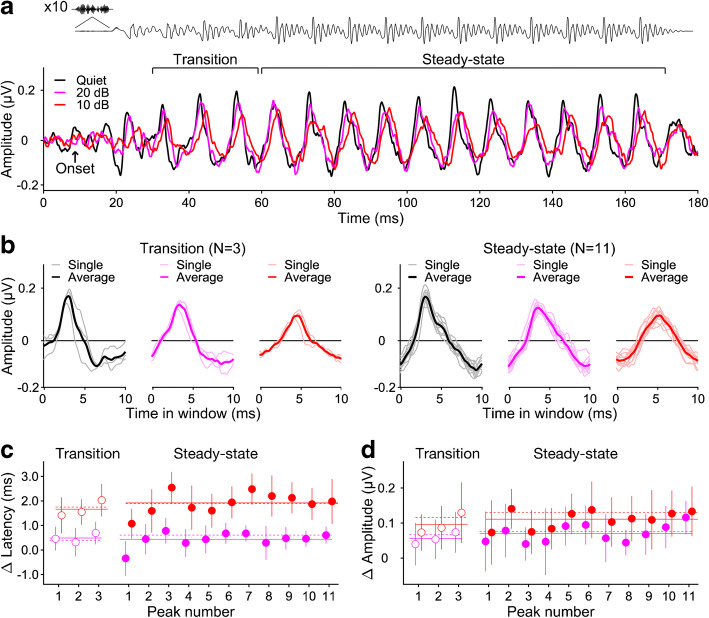
Grand-average broadband speech ABRs (*N* = 12) recorded in quiet and in background noise. **a** Overall responses, with the responses for different noise conditions overlaid (black: in quiet; pink: 20-dB SNR; red: 10-dB SNR). The stimulus waveform is shown above the response, delayed by the latency of the onset peak (8.7 ms; see arrow) to align response peaks with eliciting glottal pulses. The portion labelled ‘transition’ comprises three of the glottal pulses within the formant transition, and the ‘steady-state’ portion comprises the 11 glottal pulses that make up the following steady-state vowel. **b** Same responses as in **a**, but now shown within overlaid consecutive 10-ms windows (separately for the transition and steady-state portions). Each window contains a single response peak. The thin lines show the individual peaks for each window, and the thick lines show their average. **c**, **d** Average noise-induced latency (**c**) and amplitude (**d**) changes of each response peak as a function of peak number within the transition (left) and steady-state (right) portions. The error bars show the 95% confidence intervals (CIs) across participants. The solid horizontal lines show the average latency and amplitude changes across the individual response peaks within each response portion (corresponding to the thin lines in **c** and **d**), and the dashed horizontal lines show the latency and amplitude changes of the respective averaged response peaks (bold lines in **c** and **d**)

All offline data processing and analysis were performed in MATLAB. Responses were first bandpass-filtered between 70 and 2000 Hz using a 12-dB/oct, zero-phase Butterworth filter. Then, they were divided into ‘transition’ and ‘steady-state’ portions, corresponding to the consonant transition and steady-state vowel portions of the speech stimulus (Fig. 2a). The transition portion ranged from 30 to 60 ms post-stimulus onset and comprised three consecutive response peaks, corresponding to the second to fourth of a total of 15 glottal pulses. The steady-state portion was longer, ranging from 60 to 170 ms and comprising 11 peaks, corresponding to all remaining glottal pulses. The responses to the initial stop burst and first glottal pulse were discarded due their poor signal-to-noise ratio in the conditions with background noise. The transition and steady-state portions were each further divided into consecutive 10-ms segments, each comprising a single response peak (transition, *N* = 3; steady-state, *N* = 11; see Fig. 2b; 10 ms corresponds to the glottal-pulse period). In the broadband response, these individual peaks were affected similarly by the background noise, as confirmed statistically (see “Results”) and were thus averaged to produce a representative averaged-peak response (Fig. 2b). In the derived-band responses, the response-to-noise ratio was often too low to evaluate the individual peaks, and so, only the averaged peaks were evaluated.

A conditional automated procedure was used to determine the latencies and amplitudes of the response peaks for each condition, response portion and participant. For the broadband responses and the derived-band responses in quiet, we first found the peak (largest positive amplitude within the relevant 10-ms response time window) of the respective grand-average response across participants, and then sought the peak of each individual participant’s response within the 4-ms time window around the peak latency of the grand-average response. For the derived-band responses with background noise, the 4-ms search windows for each participant were based on that participant’s peak latency of the respective derived-band response in quiet. This was because even the grand-average responses for some of the derived-band conditions with background noise did not contain any discernible peak over and above the response noise floor.

When there is no discernible response peak, a peak latency cannot be meaningfully assessed. Therefore, before analysing the peak latencies, the peak amplitudes for each condition were first statistically compared with the amplitude of the associated response noise floor. The amplitude of the noise floor of a given response was estimated as the root-mean-square (rms) amplitude of the respective plus-minus reference (see above). As the rms amplitude of a response with a given peak amplitude depends on the response shape, rms and peak amplitudes cannot be compared directly. Thus, the noise rms amplitudes were transformed into units of peak amplitude by multiplication with a factor, *F*, derived by linearly fitting, with zero intercept, the relationship between the rms and peak amplitudes of the responses across all conditions and participants for each response portion. This yielded *F* = 0.559 (*R*^2^ = 0.819) for the transition portion and *F* = 0.496 (*R*^2^ = 0.902) for the steady-state portion. Statistical analysis of the noise rms amplitudes showed significant main effects of derived frequency band (*F*(3,253) = 17.1, *p* < 0.001) and response portion (*F*(1,253) = 116, *p* < 0.001), and a significant frequency band by response portion interaction (*F*(3,253) = 4.53, *p* = 0.004). There were, however, no significant main or interaction effects of background noise condition (all *p* ≥ 0.123), and so, the noise rms amplitudes for each frequency band condition and response portion were averaged across background noise conditions. Conditions for which the peak amplitudes did not significantly exceed the resulting transformed and averaged noise floor amplitudes (according to a paired *t* test) were excluded from the analysis of the latencies.

In addition to extracting the latencies and amplitudes of the response peaks, we also calculated the responses’ frequency spectra and stimulus-to-response correlations. The frequency spectra were calculated by applying a fast Fourier transform to the relevant response portion (30–60 ms for the transition, 60–170 ms for the steady-state). Spectral amplitudes were then calculated within 40 Hz-wide bins around the syllable fundamental frequency, F0 (100 Hz) and its second to fifths harmonics, H2 to H5 (*k*·100 Hz, *k* = 2, …, 5), the latter of which were subsequently summed. To estimate the noise floor of the response spectra, the same procedure was applied to the plus-minus references. The stimulus-to-response correlations were calculated by cross-correlating the relevant portions of the stimulus and response within the 4-ms time window around the latency of the respective averaged response peak (see above) and finding the lag where the correlation was maximal. The maximum correlation coefficients were Fisher z-transformed for normality.

### Place Mechanism Simulation

In order to quantify the potential contribution of the place mechanism to the observed noise-induced changes of the broadband speech ABR, we performed a simple simulation that implemented the place mechanism artificially using the noise effects on the derived-band response amplitudes. The simulation was performed using the averaged-peak responses for the transition and steady-state portions (see above). It was based on the fact that the broadband response is mathematically equivalent to the sum of all derived-band responses, and the assumption that the background noise changed the amplitudes, but not the temporal properties, of the derived-band responses. Thus, effectively, we assumed that the noise effects on the broadband response were caused exclusively by the place mechanism.

The simulated broadband responses in noise, *y*^(*n*)^(*t*) (where *t* is time), were calculated by reweighting the derived-band responses in quiet, *x*_*i*_^(*q*)^(*t*) (where *i* indexes the derived frequency band, < 1 kHz, 1–2 kHz, 2–4 kHz or > 4 kHz), according to their peak amplitudes in background noise, *A*_*i*_^(^^*n*^^)^, and then summing the reweighted responses: *y*^(*n*)^(*t*) = ∑_*i*_(*A*_*i*_^(*n*)^/*A*_*i*_^(*q*)^)*x*_*i*_(*t*). The division by *A*_*i*_^(*q*)^, the derived-band amplitudes in quiet, normalises the response amplitudes to unity before reweighting.

As the responses of individual participants were noisy, and the corresponding response amplitudes were associated with a high degree of variability, the simulation was instead performed with bootstrap-average responses (using 1000 different bootstrap samples). The response peak latencies and amplitudes of the bootstrap-average and simulated responses were determined in the same way as for the individual participant’s responses (see previous section).

Derived frequency bands for which the individual participants’ responses had failed to significantly exceed the response noise floor (see above) were excluded from the simulation.

### Statistical Analyses

All statistical analyses were conducted using *R* (R Core Team 2013). Individual participants’ response peak latencies and amplitudes were fitted with linear mixed-effects models (nlme package; Pinheiro et al. 2013), which included fixed effects of background noise condition (quiet, 20- and 10-dB SNR), response type (transition and steady-state) and, where applicable, derived-band condition (< 1, 1–2, 2–4 and > 4 kHz), as well as by-subjects random intercepts and fixed-factor slopes. The fixed effects were fitted with maximum likelihood (ML) estimation, and the random effects with restricted ML (REML) estimation. Random effects were omitted if they failed to produce a significant improvement in model fit. This was tested with log-likelihood ratio tests. Fixed effects were evaluated using type-III (marginal) conditional F-tests following the strategy described in Pinheiro and Bates (2000).

Normality of the data was verified by visual inspection of the quantile-quantile plots of the model residuals. Homogeneity of variance was tested by submitting the model residuals to Levene’s test (car package; Fox and Weisberg 2011). Where variance homogeneity was violated, the data were inverse-variance-weighted and the model refitted. Significant fixed effects were post hoc-tested using Holm-Bonferroni correction to control familywise error rate (lsmeans package; Lenth 2016).

## Results

Previous speech ABR studies have evaluated the latencies and amplitudes of individual response peaks, corresponding to individual glottal pulses in the stimulus waveform, and often also calculated summary response measures, such as the stimulus-to-response correlation, or the fundamental (F0) and/or harmonic peak amplitudes in the response frequency spectrum. Some of these measures were evaluated separately for the transition and steady-state portions of the response. Here, we likewise divided the responses into transition and steady-state portions, comprising the second to fourth and remaining 11 response peaks, respectively (see “Materials and Methods” and Fig. 2a). For the broadband response, we evaluated the latencies and amplitudes of both the individual peaks and the averaged peaks across each response portion (see “Materials and Methods” and Fig. 2b–d). For the derived-band responses, the response-to-noise ratio of many of the individual peaks was too low for meaningful analysis, and so, only the averaged peaks were evaluated. In addition, we also calculated the stimulus-to-response correlations, as well as the F0 and harmonic amplitudes in the response frequency spectra. These were compared qualitatively to the peak latencies and amplitudes, but were not submitted to a statistical analysis.

### Effect of Background Noise on the Broadband Speech ABR

**Fig. 3:**
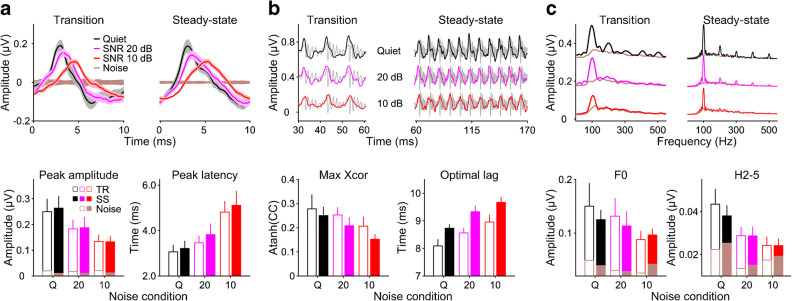
Effect of background noise on the broadband speech ABR. **a** Averaged response peaks. Top panel: Grand-average response waveforms (*N* = 12) overlaid for different noise conditions (black: in quiet; pink: 20-dB SNR; red: 10-dB SNR: red). The transition and steady-state portions are shown on the left and right, respectively. The lighter shaded margins show the standard error (SE) across participants at each time point. The brown highlight shows the estimated noise floor based on the response replicates (see “Materials and Methods”). Bottom panel: Averaged-peak amplitudes (left) and latencies (right) across participants, plotted as a function of noise condition. The parameter is the response portion (open bars: transition; filled bars: steady-state). The error bars show the 95 % CIs across participants. Small brown bars show the amplitudes of the noise floor. **b** Stimulus-to-response correlations. Top panel: Grand-average response waveforms overlaying the stimulus at the optimal correlation lag (the lag that maximises the correlation coefficient). Bottom panel: Average correlation coefficients, transformed for normality (left) and optimal correlation lags (right) as a function of noise condition. **c** Spectral amplitudes. Top panel: Grand-average response spectra. The brown lines show the spectra of the noise floor. Bottom panel: Average spectral amplitudes of the fundamental frequency (F0) (left) and the second to fifth harmonics (H2–5) (right) as a function of noise condition. The smaller brown bars show the corresponding noise floor amplitudes

Consistent with previous studies, the background noise caused the peaks in the broadband speech ABR to decrease in amplitude, increase in latency and broaden in shape (Fig. 2a, b). The size of these effects seemed to increase with increasing background noise level. For the individual peaks, the main effect of background noise was significant for both the peak latencies (transition, *F*(2,88) = 55.1, *p* < 0.001; steady-state, *F*(20,352) = 72.9, *p* < 0.001; Fig. 2c) and the peak amplitudes (transition, *F*(2,88) = 17.9, *p* < 0.001; steady-state, *F*(20,352) = 21.1, *p* < 0.001; Fig. 2d), but there was no significant interaction with peak number for either response portion (latencies *p* ≥ 0.139; amplitudes *p* ≥ 0.525), indicating that the noise effects did not significantly vary with peak number. Therefore, subsequent analyses were performed on the latencies and amplitudes of the averaged peaks for each response portion (Figs. 2c, d and 3a). Like the individual peaks, the averaged peaks showed significant effects of background noise on both their latencies (*F*(2,55) = 45.4, *p* < 0.001) and amplitudes (*F*(2,55) = 33.6, *p* < 0.001), and these effects did not interact significantly with response portion (latencies, *F*(2,55) = 1.17, *p* = 0.319; amplitudes, *F*(2,55) = 0.155, *p* = 0.856). The main effect of response portion was non-significant for the averaged-peak amplitudes (*F*(1,55) = 0.244, *p* = 0.624), but marginally significant for the latencies (*F*(1,55) = 3.77, *p* = 0.057), reflecting the fact that the averaged-peak latencies were slightly longer for the steady-state than for the transition portion (Fig. 3a, bottom right panel). This was true for all three noise conditions, with an average difference of 0.27 ± 0.14 ms.

Similar to the response peak amplitudes, the maximum stimulus-to-response correlation coefficients, and the responses’ F0 and harmonic spectral amplitudes, also decreased in the presence of background noise (Fig. 3b, c). The harmonic amplitudes seemed to be particularly strongly affected by the noise (bottom right panel in Fig. 3c), barely peaking above the noise floor at the higher noise level (10-dB SNR). Similar to the peak latencies, the optimal correlation lags increased in the presence of background noise (Fig. 3b, bottom right panel), and were longer for the steady-state than for the transition portion. The difference appeared to be considerably larger for the correlation lags than for the latencies. Smaller, and less consistent, differences between the response portions were also notable in the correlation coefficients and the F0 and harmonic spectral amplitudes, with generally smaller coefficients and amplitudes in the steady-state than in the transition portion. This difference was not evident in the peak amplitudes (see above).

### Derived-Band Speech ABRs in Quiet

**Fig. 4:**
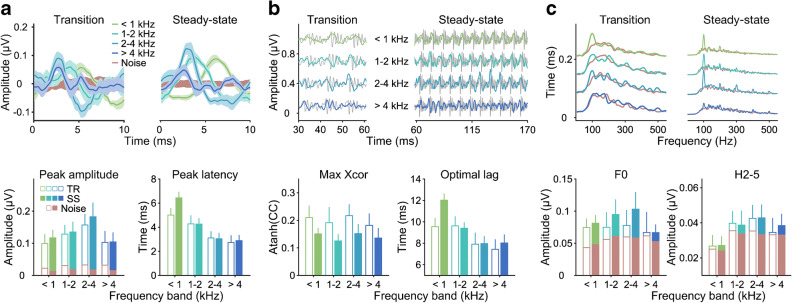
Derived-band speech ABRs in quiet. **a** Averaged response peaks for different derived frequency bands. Top panel: Grand-average response waveforms, plotted in the same way as the broadband responses in Fig. 3a. Different frequency bands are indicated by different colours (see legend). Bottom panel: Average derived-band amplitudes (left) and latencies (right) across participants as a function of derived frequency band. **b**, **c** Stimulus-to-response correlations and spectral amplitudes of the derived-band responses in quiet, plotted as for the broadband responses in Fig. 3b, c

As shown in our previous study (Nuttall et al. 2015), the derived-band responses in quiet (Fig. 4a) varied systematically with derived frequency band. Their averaged-peak latencies increased progressively with decreasing frequency band (*F*(3,77) = 62.7, *p* < 0.001), and their peaks became noticeably wider (see Fig. 4a, not tested statistically). The latencies were highly similar between the transition and steady-state portions, except in the lowest frequency band (< 1 kHz), where they were considerably longer (by 1.43 ± 0.33 ms) for the steady-state than for the transition portion, giving rise to a significant main effect of response portion (*F*(1,77) = 6.77, *p* = 0.011) and a significant response portion by frequency band interaction (*F*(3,77) = 6.05, *p* < 0.001). The averaged-peak amplitudes showed a pronounced maximum in the 2–4 kHz band, yielding a significant main effect of frequency band (*F*(3,77) = 6.26, *p* < 0.001). The main effect of response portion was not significant (*F*(1,77) = 0.203, *p* = 0.143), nor was the interaction between frequency band and response portion (*F*(3,77) = 0.392, *p* = 0.759).

The F0 and harmonic spectral amplitudes (Fig. 4c) showed a similar pattern to the peak amplitudes, although both were generally very close to the noise floor. Interestingly, whilst the F0 amplitudes exceeded the noise floor mostly in the lower-frequency bands (particularly for the transition portion), the harmonic amplitudes did so mostly in the mid- and higher-frequency bands (particularly, the 1–2 and 2–4 kHz bands). The maximum correlation coefficients were largely similar across derived frequency bands—unlike both the peak amplitudes and the spectral amplitudes. In fact, the strongest effect on the correlation coefficients seemed to be of response portion, with considerably greater correlations for the transition than for the steady-state portion. The optimal correlation lags (Fig. 4b) showed a highly similar pattern to the peak latencies, increasing progressively with decreasing frequency band. Like the latencies, the lags were mostly similar between the response portions, except in the lowest frequency band where they were considerably longer for the steady-state than for the transition portion.

### Effect of Background Noise on the Derived-Band Speech ABRs

**Fig. 5:**
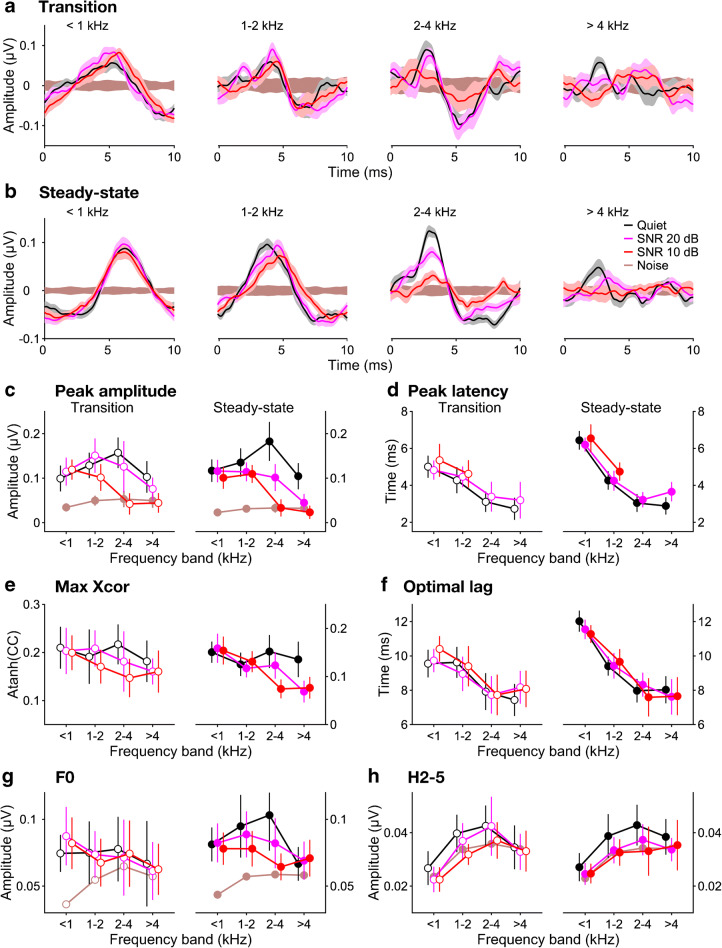
Background noise effects on derived-band speech ABRs. **a**, **b** Grand-average waveforms (*N* = 12) of averaged response peaks, overlaid for different noise conditions and plotted as for the broadband responses in Fig. 3a. Panels **a** and **b** show the transition and steady-state portions, respectively, and the different axes with each panel show the different frequency bands (ordered from low to high; see axis titles). **c**, **d** Average derived-band amplitudes (**c**) and latencies (**d**) across participants as a function of derived frequency band. The parameter is the noise condition (same colours as in **a** and **b**). The results for the transition and steady-state portions are shown on the left and right, respectively. The error bars show the 95% CIs across participants. The brown symbols and lines in panel **c** show the amplitudes of the noise floor for each frequency band. **e**, **f** Average stimulus-to-response correlations across participants (**e**) and optimal correlation lags (**f**). **g**, **h** Average F0 (**g**) and harmonic (H2–5) amplitudes (**h**) across participants. The brown symbols and lines show the corresponding noise floor amplitudes

The effect of background noise on the derived-band responses was markedly different between frequency bands (Fig. 5a, b). Whilst the lower two frequency bands (< 1 and 1–2 kHz) were relatively unaffected by the noise, the higher two bands (2–4 and > 4 kHz) both showed pronounced decreases in averaged-peak amplitude (Fig. 5c). This was confirmed by a significant two-way interaction between background noise condition and derived frequency band (*F*(6,253) = 12.5, *p* < 0.001). This pattern did not differ significantly between the response portions (three-way interaction between response portion, background noise condition and frequency band: *F*(6,253) = 0.922, *p* = 0.475). Post hoc analysis confirmed that the effect of background noise was significant for the higher two frequency bands (both *p* ≤ 0.002), but not for the lower two bands (both *p* ≥ 0.126). In fact, at the higher of the two noise levels (20-dB SNR), the noise effect on the higher two bands was so strong that their averaged-peak amplitudes failed to reach significantly above the response noise floor (see “Materials and Methods”). This made it impossible to extract meaningful peak latencies for these conditions. As a result, the latencies (Fig. 5d) could not be combined into a single statistical model for both noise levels. Instead, they were submitted to two separate models, which combined the available frequency bands for each of the two noise levels (all four bands for 10-dB SNR; only the lower two bands for 20-dB SNR) with the corresponding frequency bands for the in-quiet condition.

**Fig. 6:**
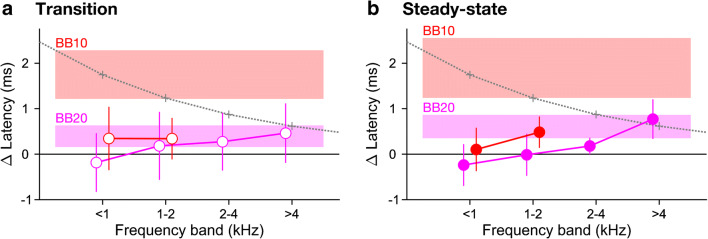
Noise-induced *changes* in derived-band latency relative to the quiet condition. Panels **a** and **b** show the transition and steady-state portions, respectively. The coloured symbols show the average latency changes across participants as a function of derived frequency band. The error bars show the 95% CIs. The parameter is the noise level (pink: 20-dB SNR; red: 10-dB SNR). The grey lines and symbols show the maximal within-band latency changes that could theoretically arise within each frequency band, estimated using the travelling-wave delay measurements by Strelcyk et al. (2009). The rectangular coloured areas labelled ‘BB20’ and ‘BB10’ show the 95% CIs of the noise-induced changes in broadband response latency for each noise level. Latency shifts could not be assessed and are thus not shown for conditions where the response peaks did not reliably exceed the noise floor across participants

Neither of the models revealed any significant effects of background noise. Unlike the latencies of the broadband response, which had shown highly significant main effects of noise condition at both noise levels (see above), the derived-band latencies showed no main effect of noise condition at either noise level (both *p* ≥ 0.105). There were also no significant interactions with noise condition, except for a marginal interaction between noise condition and derived frequency band at the lower noise level only (*F*(3,165) = 2.43, *p* = 0.067). This reflects a trend for the noise effect at the lower noise level to vary with frequency band (Fig. 6): the effect was slightly negative for the lowest band (latency decrease compared with the quiet condition), but then increased towards higher bands until, for the highest band, it just reached significance (*p* = 0.023).

The correlation coefficients (Fig. 5e) showed a similar pattern as the peak amplitudes, albeit with much larger relative errors. Specifically, in the lower two frequency bands, the correlation coefficients were little affected by the presence of background noise, even at the higher of the two noise levels, whereas, in the higher two bands, the coefficients were considerably reduced. Like the peak latencies, the correlation lags showed little or no effect of background noise (Fig. 5f). If anything, the noise effects on the lags were smaller than those on the latencies and were absent for the higher two frequency bands and the higher noise level, where peak latencies could not be assessed.

In the steady-state portion, the F0 spectral amplitudes, again, showed a similar pattern as the peak amplitudes (Fig. 5g, right panel). In the transition portion, the F0 amplitude mostly failed to clear the noise floor sufficiently to be able to observe a notable effect of background noise. Only in the lower two bands (< 1 and 1–2 kHz) did the transition F0 amplitudes significantly exceed the noise floor, and, consistent with the peak amplitudes, these showed little or no effect of background noise. The harmonic spectral amplitudes (Fig. 5h), similarly, showed little margin above the noise floor for all but the middle two frequency bands (1–2 and 2–4 kHz), particularly in the transition portion. Within this small margin, the pattern of background noise effects appeared to be consistent with that observed for the peak amplitudes.

### Place Mechanism Simulation

The results so far have shown substantial increases in the peak latency, and decreases in the peak amplitude, of the broadband speech ABR in the presence of background noise. In contrast, whilst background noise caused a large reduction in the peak amplitudes of the higher-frequency derived-band speech ABRs, it generally had little effect on the derived-band peak latencies (see Figs. 5 and 6). This general pattern was mirrored by both the stimulus-to-response correlations and the F0 and harmonic spectral amplitudes, with the correlation coefficients and spectral amplitudes broadly matching the pattern of the peak amplitudes and the optimal correlation lags broadly matching the pattern of the peak latencies.

The findings for the derived-band responses suggest that the background noise effects on the broadband speech ABR contained substantial contributions of the place mechanism. In order to quantify these contributions, we simulated the effects based on the assumption that they were *exclusively* generated by the place mechanism. The simulation used the derived-band speech ABRs in quiet and their peak amplitudes in noise (see “Materials and Methods”).

**Fig. 7:**
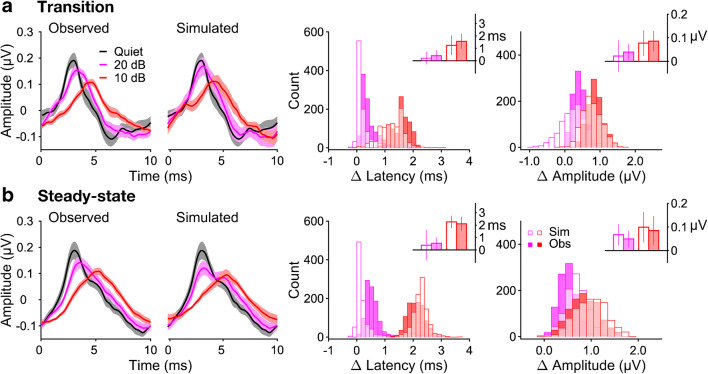
Simulation of the place effect on the broadband speech ABR. **a**, **b** Left two axes: Observed (left) and simulated (middle) broadband responses for the different noise conditions. Panels **a** and **b** show the transition and steady-state portions, respectively. The solid lines show the bootstrap grand-average responses, and the lighter shaded margins show the bootstrap 95% CIs at each time point (both based on 1000 bootstrap samples). Note that, in quiet (black lines), the simulated responses are identical to the observed ones. Right two axes: Bootstrap histograms of the simulated (open bars) and observed (filled bars) latency and amplitude changes for the two noise levels (pink: 20-dB SNR; red: 10-dB SNR). The latency changes are shown on the left, and the amplitude changes on the right. The insets show the bootstrap-average latency and amplitude changes. The error bars show the corresponding bootstrap 95% CIs

Figure 7 shows that, at least to the first order, the simulation produced a good approximation of the broadband speech ABR waveform at both noise levels and for both response portions (left panels), and also produced a good approximation of the noise-induced changes in the response latencies and amplitudes (right panels). At the lower noise level, the latency changes were slightly underestimated for both response portions, and at the higher noise level, the changes were slightly underestimated for the transition portion, but overestimated for the steady-state portion. Similarly, for the response amplitudes, the noise effects were generally underestimated for the transition portion, but overestimated for the steady-state portion. Generally, however, the differences between the simulated and observed effects were relatively small, particularly in relation to the large inter-individual variability (reflected by the bootstrap standard error; see insets). The differences ranged from 0.15 to 0.32 ms in the case of the latencies, and from 0.083 to 0.18 μV in the case of the amplitudes.

## Discussion

This study aimed to investigate whether noise-induced changes in the broadband speech ABR, interpreted by several previous studies to represent a *neural* correlate of SiN difficulty, could be confounded by the *cochlear* place mechanism. In the place mechanism, faster and more synchronised response contributions from higher-frequency cochlear regions are specifically reduced by background noise, leading the aggregate (broadband) response to become reweighted towards slower and less-synchronised contributions from lower-frequency cochlear regions. To test this, we measured both broadband and derived-band speech ABRs in quiet and in two levels of uniformly exciting noise.

Consistent with previous results (Anderson et al. 2010; Cunningham et al. 2001; Parbery-Clark et al. 2009), the broadband response showed significant and sizeable increases in peak latency, and reductions in peak amplitude, in the presence of both noise levels. In contrast, the noise effects on the derived-band responses were mostly limited to the peak amplitudes, with only small and mostly non-significant effects on the latencies. Importantly, the derived-band amplitude effects were highly specific in frequency, with substantial amplitude reductions in the higher-frequency bands—in some cases, to below the response noise floor—but little or no amplitude changes in the lower-frequency bands. The noise effects on the stimulus-to-response correlations and response spectral amplitudes at the stimulus fundamental frequency (F0) and harmonics were qualitatively similar to those observed for the response amplitudes and latencies, although, invariably, the signal-to-noise ratio of these measures was much lower, and the relative error was much larger, than for the amplitudes and latencies. Overall, these results suggest that the noise indeed caused a reweighting of the broadband speech ABR, and that this contributed substantially to the observed noise effects on its properties. To test this further, we performed a simulation of the place mechanism by modelling the broadband responses in noise as a sum of the reweighted derived-band responses in quiet. Within the variability of the data, the simulated effects largely accounted for the full extent of the observed effects, leaving little scope for any neural effects—at least for our chosen experimental conditions and our group of young and normal-hearing participants.

The noise-induced latency changes of the derived-band speech ABRs were mostly non-significant and generally much smaller than those of the broadband response. At the lower noise level, however, where the latency changes could be evaluated for all frequency bands, there was a marginally significant tendency for the latency changes to increase towards higher-frequency bands, such that, in the highest band, the change was both significant and comparable in size with the corresponding broadband latency change (note, however, that, as the amplitude of the highest frequency band was strongly reduced, even at the lower noise level, its latency change cannot have contributed substantially to the corresponding broadband latency change). Under the assumption that this was not a false positive result, this raises the question whether the change was mediated by neural mechanisms, or whether it too, like the bulk of the broadband latency changes, was caused by the cochlear place mechanism. Within the derived frequency bands, a place effect would shift the response peak downwards within the band’s frequency range. If the frequency range covers a large range of cochlear travelling-wave delays, the resulting increase in response latency can be sizeable. In the current study, the maximum possible place-based derived-band latency changes, estimated using the travelling-wave delay measurements by Strelcyk et al. (2009), numerically exceeded the observed latency changes for all derived frequency bands (see grey lines and symbols in Fig. 6), and so, a place-based explanation cannot be excluded even for the significant change in the highest frequency band at the lower noise level. However, Burkard and Hecox (1987), using a click as stimulus, observed derived-band latency changes that could not be explained based on the place mechanism. Thus, at least under certain circumstances, noise-induced latency changes of ABRs may be at least partly caused by neural mechanisms. A possible candidate mechanism that has been suggested previously is neural adaptation (Burkard and Hecox 1983a). Alternatively, neurally mediated noise effects on ABRs may be caused by top-down influences from higher brain stages (Presacco et al. 2016).

Previous studies of noise effects on the speech ABR have mostly (e.g. Parbery-Clark et al. 2009; Anderson et al. 2010; Song et al. 2011), although not exclusively (Cunningham et al. 2001), used babble noise as background noise, whereas the current study used uniformly exciting noise. Babble noise has the same high-frequency roll-off as speech (about − 6 dB/oct), whereas uniformly exciting noise has a somewhat shallower high-frequency roll-off (about − 3 dB/oct) and thus contains relatively more high-frequency energy. This means that the signal-to-noise ratio decreased towards higher frequencies (see Fig. 1c). Previous findings with non-speech ABRs suggest that this difference may have caused a greater place effect in the current compared with the previous studies. Using 1- and 4-kHz tone pips as stimulus, and white noise as background noise, Burkard and Hecox (1983b) found a much greater place effect for the 1- than 4-kHz tone pip, suggesting that the size of the place effect depends on the relative high-frequency content of the stimulus and noise.

However, the same study (Burkard and Hecox 1983b) also demonstrated that a significant place effect can occur even when the stimulus and noise contain the same amount of high-frequency energy, as was the case in most of the previous speech ABR studies. Using a click as stimulus and white noise as background noise, both of which have a flat high-frequency spectrum, Burkard and Hecox nevertheless found greater noise-induced amplitude reductions of the higher- than lower-frequency derived-band responses. This suggests that, whilst the place effect may have been greater in the current than the previous speech ABR studies, it is unlikely that the previous studies were completely unaffected by it.

Burkard and Hecox’s (1983b) finding with the click stimulus demonstrates that ABR contributions from higher-frequency cochlear regions are inherently more susceptible to background noise than contributions from lower-frequency regions. Perceptual effects of noise, by contrast, are largly constant across frequencies or, if anything, greater at lower frequencies (Hawkins and Stevens 1950). This suggests that noise effects on perception versus ABRs are based on different underlying mechanisms. Under the common and well-established assumption that ABRs are related linearly with their underlying neuroelectric sources (Møller 2007), a ‘line-busy’ effect of noise, whereby the stimulus- and noise-related responses merely mix without affecting one another’s size (Delgutte 1990), should not cause any measurable changes in the stimulus-related ABR. In contrast, a suppressive effect of noise, whereby the noise-related response reduces the size of the stimulus-related response, should manifest as a reduction in the stimulus-related ABR amplitude. Thus, the absence of noise effects on lower-frequency derived-band responses suggests that, at lower frequencies, noise effects are predominantly line-busy effects, whereas, at higher frequencies, where noise causes large reductions in ABR amplitude, noise effects are strongly suppressive. Suppressive noise effects at higher frequencies may be caused by neural adaptation (Sumner and Palmer 2012) or two-tone suppression (Delgutte 1990).

Whilst the stimulus-to-response correlations and the response frequency spectra showed qualitatively similar effects to the response amplitudes and latencies, they also revealed some interesting complementary results, which shed light on the factors that may influence these measures. Previous studies of speech or complex ABRs have taken the amplitudes of the fundamental (F0) and harmonic peaks in the response frequency spectra as measures of the neural encoding accuracy of the corresponding frequencies within the stimulus frequency spectrum. For instance, it has been suggested that larger F0 and harmonic amplitudes may reflect superior ability to process pitch and timbre cues (Anderson et al. 2011; Cunningham et al. 2001; Parbery-Clark et al. 2009, 2012; Song et al. 2011; Strait et al. 2012), or temporal envelope and fine structure cues (Ruggles et al. 2012). This interpretation, however, does not seem consistent with our finding, for the derived-band responses in quiet, that the higher-frequency bands (in particular, the 2–4 kHz band) contained larger harmonic amplitudes than the lower-frequency bands, even though the relevant frequencies (200–500 Hz) were all contained well within frequency range of the lowest band (< 1 kHz). This suggests that, rather than reflecting the neural encoding accuracy of the stimulus spectral features, ABR spectra predominantly reflect the shapes of their cochlear response contributions, which, in turn, are determined by the ringing times of the respective cochlear filters: higher-frequency filters are broader and thus have shorter ringing times, yielding sharper ABR contributions with larger harmonic amplitudes. This interpretation is supported by our observation that the harmonic amplitudes in the broadband response were practically abolished at the higher noise level, where the higher-frequency response contributions with larger harmonic amplitudes were also abolished. Moreover, it is also consistent with the fact that frequency decomposition (Fourier analysis) is a linear process and, thus, that the spectrum of the broadband response, which equals the sum of the derived-band responses, should equal the sum of the spectra of the sum of the derived-band responses. Finally, it also accords with the finding by Janssen et al. (1991) that frequency-following responses elicited by low-frequency pure tones are dominated not by low-frequency, but by higher-frequency, response contributions.

Unlike response frequency spectra, stimulus-to-response correlations measure directly the similarity between a given ABR and the eliciting stimulus, and, like the spectra, they too are widely deemed a measure of the neural encoding accuracy of the stimulus (Cunningham et al. 2001; Parbery-Clark et al. 2009; Parbery-Clark et al. 2011; Parbery-Clark et al. 2012). However, again, some aspects of the current results do not seem compatible with this interpretation. Unlike the responses’ peak and spectral amplitudes, the stimulus-to-response correlations of the derived-band responses in quiet were comparatively constant across derived frequency bands, but showed a substantial difference between response portions, with, surprisingly, greater correlations for the transition than for the steady-state portion. According to the idea that stimulus-to-response correlations reflect neural encoding accuracy, this would imply that the smallest and noisiest responses—the transition responses for the lowest and highest frequency bands—encoded the stimulus as well as, or better than, the largest and most robust responses—the steady-state responses for the mid-frequency bands. This, of course, is implausible and thus calls for caution in interpreting stimulus-to-response correlations too literally and without consideration of possible mathematical or physiological confounds.

### Summary

The present findings show that interpretations of noise-induced changes in the speech ABR—in line with those of non-speech ABRs—are potentially confounded by cochlear effects relating to the response’s predominant place of origin. The contribution of the place mechanism will depend on the acoustic properties of the speech stimulus and background noise, but is unlikely to be eliminated by simply matching their spectra. The derived-band technique provides a useful tool for quantifying the place effect under given conditions, and thus assessing the ‘true’ size of any neurally generated effects. It is possible that such neural effects may be greater in participants with SiN deficits, such as children with speech and language impairment or older adults, compared with young normal controls as tested in the current study.
